# Drug–Drug Interactions in Nosocomial Infections: An Updated Review for Clinicians

**DOI:** 10.3390/pharmaceutics16091137

**Published:** 2024-08-28

**Authors:** Sorina Hîncu, Miruna-Maria Apetroaei, Gabriela Ștefan, Anca Ionela Fâcă, Andreea Letiția Arsene, Beatrice Mahler, Doina Drăgănescu, Adriana-Elena Tăerel, Emilia Stancu, Lucian Hîncu, Andreea Zamfirescu, Denisa Ioana Udeanu

**Affiliations:** 1 Faculty of Pharmacy, Carol Davila University of Medicine and Pharmacy, 6, Traian Vuia Street, 020956 Bucharest, Romania; sorina.hincu@gmail.com (S.H.); gabriela.stefan@rez.umfcd.ro (G.Ș.); anca-ionela.faca@drd.umfcd.ro (A.I.F.); andreea.arsene@umfcd.ro (A.L.A.); doina.draganescu@umfcd.ro (D.D.); adriana.taerel@umfcd.ro (A.-E.T.); emilia.stancu@umfcd.ro (E.S.); lucian.hincu@umfcd.ro (L.H.); denisa.udeanu@umfcd.ro (D.I.U.); 2 Fundeni Clinical Institute, 258, Fundeni Street, 022328 Bucharest, Romania; 3 Marius Nasta Institute of Pneumophthisiology, 90, Viilor Street, 050159 Bucharest, Romania; beatrice.mahler@umfcd.ro; 4 Faculty of Medicine, Carol Davila University of Medicine and Pharmacy, 8, Eroii Sanitari Street, 050474 Bucharest, Romania; 5 Faculty of Midwifery and Nursing, Carol Davila University of Medicine and Pharmacy, 8, Street, 050474 Bucharest, Romania; andreea.zamfirescu@umfcd.ro

**Keywords:** drug–drug interactions, nosocomial infections, drug–drug interaction management, personalised therapy, antibiotic therapy, pharmacotherapeutic considerations, therapeutic drug monitoring, antibiotic pharmacokinetics, antibiotic pharmacodynamics

## Abstract

Prevention, assessment, and identification of drug–drug interactions (DDIs) represent a challenge for healthcare professionals, especially in nosocomial settings. This narrative review aims to provide a thorough assessment of the most clinically significant DDIs for antibiotics used in healthcare-associated infections. Complex poly-pharmaceutical regimens, targeting multiple pathogens or targeting one pathogen in the presence of another comorbidity, have an increased predisposition to result in life-threatening DDIs. Recognising, assessing, and limiting DDIs in nosocomial infections offers promising opportunities for improving health outcomes. The objective of this review is to provide clinicians with practical advice to prevent or mitigate DDIs, with the aim of increasing the safety and effectiveness of therapy. DDI management is of significant importance for individualising therapy according to the patient, disease status, and associated comorbidities.

## 1. Introduction

Nosocomial infections are those infections acquired during the healthcare process that are not present at the time of hospital admission and occur in all healthcare settings, including hospitals, outpatient departments, chronic care hospitals, and after discharge [1]. Annually, 24% of patients suffer from nosocomial sepsis, while 52.3% of them die in intensive care wards. The mortality rate doubles to triples when the patient acquires a multidrug-resistant strain [2]. Approximately 3.5 million nosocomial infections exist in the European Union and the European Economic Area. All these result in over 90,000 fatalities and over 2.5 million lifetime disabilities. In this region, it is estimated that nosocomial infections have a higher mortality rate than the sum of cases of influenza or tuberculosis [3].

Drug–drug interactions (DDIs) occur when the effects of one drug are altered due to the simultaneous administration of other medications. In addition to potential DDIs, which may occur prior to administration, DDIs may be pharmacokinetic or pharmacodynamic [4].

Current approaches to drug treatment of multiple comorbidities are based on the use of multiple drugs from different classes and have led to the development of two associated challenges: multimorbidity and polypharmacy [5]. Moreover, a recent meta-analysis showed that patients with multiple comorbidities are more likely to develop nosocomial infections [6]. Thus, in addition to the polypharmacy already present in this category of patients, a new therapeutic plan is being added, consisting of one or more pharmacologically active substances to treat the infection caused by the pathogenic germ. As the number of therapeutic agents administered to a patient increases, so does the risk of DDIs [7].

**Figure 1:**
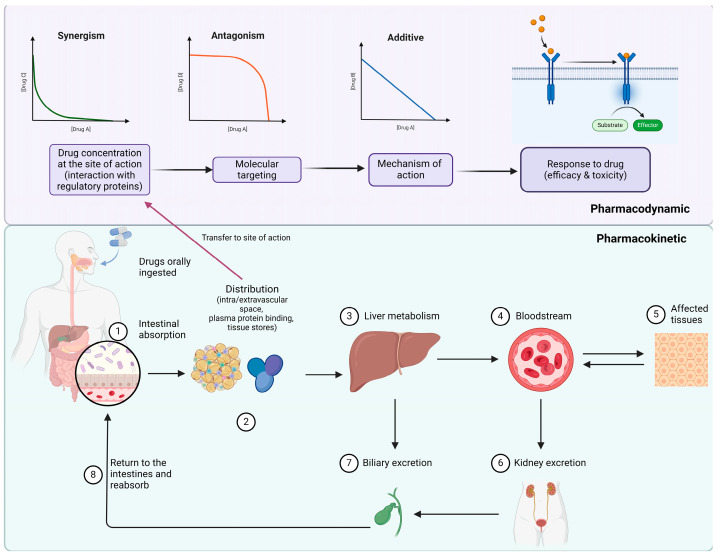
Pharmacokinetic and pharmacodynamic DDIs (created with Biorender.com, accessed on 8 June 2024).

Pharmacokinetic DDIs occur when one drug alters the way in which the other drug administered is absorbed, distributed, metabolised, or eliminated. These might result in either increased or decreased levels of the drug in the bloodstream [8]. Pharmacodynamic DDIs emerge when another co-administered drug alters the pharmacological impact of one drug [9]. Combining two medications can be synergistic when the combined effect is greater than the sum of the two effects; convergently, the interaction is antagonistic when the combined effect is less than the sum of the two effects. Suppression also refers to a high intensity of antagonism where the impact of one drug counteracts the effects of the second drug [9,10]. Figure 1 represents a visual overview of the pharmacokinetics and pharmacodynamics of drugs in the body.

Due to the manageable nature, adverse reactions resulting from DDIs can be prevented by continuous monitoring of the patient or by substituting the drug involved with another prescription when possible; however, to mitigate the risks associated with DDIs and increase the safety of the drug regimen, it is imperative that clinicians evaluate prescribed regimens and identify those combinations [11,12]. Juurlink et al. calculated that at least 3.3%, 2.3%, and 7.8% of hospitalisations of elderly patients could be avoided if drug interactions were evaluated in cases of hypoglycaemia, digoxin toxicity, and hyperkalaemia, respectively [13].

It is important to mention that not all DDIs are detrimental and that certain drugs may have increased absorption in the presence of food [14], or may have increased bioavailability when co-administered with other drugs that modulate specific enzymatic pathways [15].

Polypharmacy refers to the use of multiple drug regimens and is a significant public health issue. Polypharmacy is determined by the number of drugs used in a given time frame [16]. In a multicentre study, Kuscu et al. found that patients with potential antimicrobial-containing DDIs had a more extensive pharmacotherapeutic regimen of many antimicrobial and chronic disease medications than others. It has also been shown that the incidence of DDIs with antimicrobials increases closely with the number of antibiotics administered. Thus, the authors concluded that the need to simplify antibiotic therapy is justified [17].

In a retrospective study conducted by Wang et al., 42.5% of nosocomial infections acquired by the 15,588 patients included in the study were multidrug-resistant strains [18]. In another analysis conducted by Yang et al., 25.7% out of 1392 nosocomial infection cases were identified as multidrug-resistant bacteria [19]. A recent meta-analysis reported that 32.8% of hospital-acquired *Klebsiella pneumoniae* was multidrug resistant [20]. To avoid infections caused by multidrug-resistant Gram-negative bacteria, a combination treatment is recommended. As a potential treatment option, novel combinations are being advanced more and more. These combinations consist of antibiotics in conjunction with medications that lack antibiotic activity or antibiotics in conjunction with other antibiotics [21]. Nevertheless, this raises another concern—are DDIs going to gain a higher prevalence in the effort to treat multidrug-resistant strains?

In this narrative review, we aim to provide an in-depth analysis of the DDIs that can occur in nosocomial settings. Considering the need for precise guidelines with robust application directions, this research aims to add mechanistic explanations and potential directions for the efficient management of DDIs in healthcare-associated infections.

## 2. Clinically Relevant DDIs in Nosocomial Settings

Clinicians’ sustained efforts to optimise drug regimens should be based on minimising DDIs. In general, pharmacodynamic DDIs can be appreciated by avoiding drug combinations that show comparable adverse effects. In contrast, pharmacokinetic DDIs can be avoided by substituting other options when drug combinations are metabolised by the same P450 isoenzyme or when a drug is a known inhibitor or inducer of these enzymes. Similarly, substrate combinations for the P-glycoprotein efflux pump require additional caution [15]. There are a wide variety of factors that predispose patients to acquiring multidrug-resistant nosocomial germs, including inadequate use of broad-spectrum antibiotics, use of pharmacotherapeutic regimens that have increased potential for drug interactions, sepsis, and a recent hospitalisation for more than five days [6,22,23].

**Figure 2:**
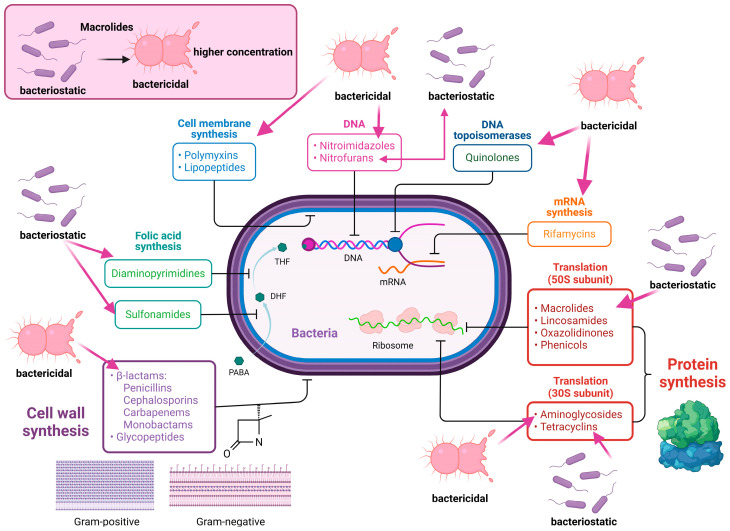
Antibiotic therapy mechanisms of action relevant to nosocomial settings (created with Biorender.com, accessed on 8 June 2024).

However, in order to understand how DDIs emerge, the mechanisms of action of antibiotics commonly used in the therapy of nosocomial infections are of particular importance. Figure 2 illustrates the mechanisms of action for the most frequently prescribed antimicrobials.

### 2.1. β-Lactam Antibiotics

β-lactam antibiotics are the most prevalent class prescribed in hospitals and are pivotal for both empirical and targeted therapy. Due to their safety profile at the recommended doses, the use of drug therapy monitoring has not been widely implemented [24]. Therefore, the need to revise the common DDIs for this class is justified.

Penicillins have a chemical structure that consists of a β-lactam ring bonded to a thiazolidine ring. All penicillins have a carboxyl group at position three of their bicyclic structure and a dimethyl group at position four. The 6-aminopenicillanic acid (6-APA) core is formed by attaching an amino group with the β-lactam ring at the C-6 position. Except for penicillin G and penicillin V, all penicillins currently used in clinical practice are derived from 6-APA by attaching different substituents to the 6-amino group [25]. Penicillins exhibit varying degrees of protein binding, with cloxacillin and dicloxacillin having protein binding values over 90%; however, piperacillin and clavulanic acid are exceptions as they are not absorbed orally. For most of the other medications in this category, gastrointestinal absorption exceeds 50% [26]. Penicillins exhibit a short half-life, ranging from around 0.5 to 1.5 h, which may vary depending on the specific molecule. Due to their solubility in water, these drugs are efficiently eliminated through the urinary system [27]. Benzathine penicillin G exhibits a prolonged apparent half-life of over 336 h, during which the antibiotic continues to enter the bloodstream for almost 30 days after one intramuscular injection [28].

Cephalosporins have favourable distribution throughout several bodily fluids, and the main route of elimination is through the kidneys. Notable exceptions are cefpiramide and cefoperazone, mainly eliminated through the bile. On the other hand, ceftriaxone is eliminated through a combination of renal and non-renal pathways [29]. Cephalosporins with a molecular weight below 450 are only excreted in the bile at a rate of less than 15% of the administered dose; however, cephalosporins with a molecular weight above 450 show a recovery rate in the bile ranging from 15% to 100%. Furthermore, the primary route for their removal is by elimination into bile and/or urine, with limited metabolic processes occurring within the body [26]. Cephalosporins are generally excreted quickly, with plasma half-lives ranging from 1 to 2 h. The exceptions include cefonicid, which has a half-life of 4.4 h; cefpiramide, which has a half-life of 5.0 h; cefotetan, which has a half-life of 3.5 h; and ceftriaxone, which exhibits the longest half-life, measuring 8.5 h [30].

Renal dihydro peptidase-1 metabolises carbapenems. Thus, in order to ensure the effectiveness of imipenem, it is necessary to administer it together with a dihydro peptidase-1 inhibitor. Meropenem, ertapenem, and doripenem have greater stability to this enzyme than other carbapenems. Carbapenems are primarily excreted through the kidneys; therefore, it is important to adjust the dosage for patients with impaired renal function. The half-life of most carbapenems is approximately 1 h, except for ertapenem, which has a half-life of 3.8 h. This longer half-life enables ertapenem to be administered once daily. Carbapenems exhibit individual variability in their pharmacokinetic characteristics, particularly in critically sick patients and those undergoing renal replacement treatment [31].

The typical serum elimination half-life of aztreonam is 2 h in individuals with normal kidney function. The protein binding rate is 56%. Approximately 66% of the drug is excreted in the urine without undergoing any changes, while 3.1% to 6.9% is removed through the kidneys as SQ26992, which is the primary metabolic byproduct of the drug. Based on urinary excretion data, the apparent half-life of SQ26992 is ten times longer than that of aztreonam. Although the site of synthesis of SQ26992 is unknown, it is produced by the process of β-lactam ring hydrolysis. The penicilloate metabolites of penicillins undergo a slower process of metabolism compared to their original molecules [32].

Table 1 illustrates clinically relevant DDIs for β-lactam antibiotics.

The inoculum effect is significant when patients acquire severe infections. It is frequently described as a decrease in antimicrobial activity (shown by a substantially higher minimum inhibitory concentration value) when the number of microorganisms used for testing exceeds the standard inoculum size. An analysis found that cephalosporins and β-lactam/β-lactamase inhibitor combinations consistently exhibited inoculum effects in laboratory tests, while carbapenems were less affected by inoculum size [48]. However, it is worth mentioning that among the various β-lactams, only carbapenems have demonstrated a post-antibiotic effect on Gram-negative bacteria. This may account for a shorter duration during which the drug concentration remains above the minimum inhibitory concentration needed for effective bactericidal activity compared to other β-lactam antibiotics. The post-antibiotic effect may be caused by the extended or irreversible acylation of penicillin-binding proteins when combined with β-lactam antibiotics. Conversely, the penicillin-binding proteins can undergo a significantly delayed deacylation reaction, which may occur gradually and lead to the reactivation of their enzymatic activity [49].

Furthermore, antibiotic treatment with β-lactams combined with probenecid is associated with improved results against gonococcal infections. Pk investigations have shown that the use of probenecid may have broad clinical applications [50].

It is also important to mention that imipenem is the drug most often involved in the occurrence of epileptic seizures in the carbapenem class. Administration of imipenem without cilastatin increases nephrotoxicity and neurotoxicity [51]. Imipenem/cilastatin—if administered in the correct dose for the treatment of severe nosocomial infections in critically ill patients with central nervous system disorders—can provide a safe therapeutic option; however, data on the use of this combination in meningitis are still limited, so this pharmacotherapeutic indication should be approached with caution [52].

### 2.2. Macrolides

Although antibiotics are used as a first-line therapy in infectious diseases caused by bacteria, the macrolide class presents additional therapeutic benefits, such as immunomodulatory effects. Moreover, recent data have highlighted the potential application of macrolides in the treatment of inflammatory airway diseases [53].

Macrolides possess lipophilic properties and display extensive distribution throughout the bloodstream and many tissues. After entering the bloodstream, macrolides have a strong affinity for binding to alpha-1-acid glycoprotein (AGP). Erythromycin exhibits a plasma protein binding affinity of around 70–80% to alpha-1-acid glycoprotein (AGP) [53]. Nevertheless, while azithromycin is 93% free in the plasma, it is only 16% unbound in the liver [54]. Macrolides have the ability to accumulate in phagocytes, which in turn carry the medication to the location of the infection. The concentrations of clarithromycin and azithromycin in phagocytes are 400-fold and 800-fold higher than those seen in the serum, respectively. The concentration of macrolides in tissues is 50 times higher than in the plasma, and macrolides have a particular affinity for the spleen, liver, lungs, and kidneys [54,55]. Erythromycin has a half-life of 1 to 2 h. Spiramycin, erythromycin stearate, the mercaptosuccinate salt of propionyl erythromycin, and rosaramicin have intermediate half-lives of approximately 7, 6.5, 5, and 4.5 h, respectively. Azithromycin has a high half-life value of 41 h. The liver is the primary pathway by which substances are removed from the body. Renal elimination occurs, although it only contributes minimally to overall clearance, as indicated by the low values of renal clearance. The impact of renal insufficiency or hepatic illness on macrolide pharmacokinetics is typically not clinically significant, so no dose adjustment is required for these patients [56,57].

Table 2 illustrates the most relevant DDIs of macrolides.

Macrolides are bacteriostatic antibiotics as they mainly inhibit the bacterial protein synthesis process. However, at higher concentrations, they show bactericidal effects (Figure 2) [72]. In murine models of acute infections, macrolides reduced apoptosis of circulant lymphocytes and the production of proinflammatory cytokines by circulating alveolar macrophages and monocytes. They also inhibit quorum-sensing proteins of the microorganism *Pseudomonas aeruginosa* from being expressed in the genome [73]. This is of increased clinical relevance as *P. aeruginosa*, one of the most common aetiological agents involved in nosocomial infections, uses chemical signals for intercellular communication. This phenomenon is called “quorum sensing” and provides the bacteria with the ability to estimate population density and synchronise their behaviour in response to fluctuations in cell densities [74]. A very recent meta-analysis evaluated how macrolides affect therapeutic outcomes in community-acquired pneumonia; in summary, when macrolides were included in the therapeutic plan, they reduced 30-day mortality by 35% and increased infection resolution by 23% compared to the control group [75].

### 2.3. Fluoroquinolones

Over the years, the fluoroquinolone class has presented multiple compounds, the first generation being already out of clinical use. At present, some drugs are constrained by inadequate pharmacokinetic characteristics or unmanageable adverse effects, such as grepafloxacin, clinafloxacin, and temafloxacin [76]. Table 3 illustrates clinically relevant DDIs for the fluoroquinolone class.

After oral administration, fluoroquinolones are quickly absorbed and extensively disseminated throughout the body. Their metabolic routes involve glucuronidation, N-oxidation, and demethylation. The primary pathway for elimination is through the kidneys, with a small amount excreted through the bile. The elimination half-lives of the quinolones range from 1.5 to 16 h [92].

As the treatments of nosocomial infections advance, the pharmacotoxicologic profile of fluoroquinolones in children is a factor that continues to contribute to their clinical limitations for children, and as ongoing changes in microbial resistance constantly pose barriers, correct and appropriate prescribing and utilisation practices of fluoroquinolones are essential for effective therapy. Clinicians should pay particular attention to adverse reactions and DDIs in the fluoroquinolone class [93]. Since 2024, the Medicines and Healthcare products Regulatory Agency (UK) has mandated that systemic fluoroquinolones should only be indicated when there are no other therapeutic alternatives [94].

### 2.4. Aminoglycosides

Aminoglycosides are well-characterised compounds that are effective against Gram-negative bacteria. Although their use has declined markedly since their discovery, they are still characterised as molecules of paramount importance as they have remained effective against many resistant germs [95]. However, this class represents a therapeutic option with multiple interactions and requires particular attention when administered in nosocomial settings (Table 4).

Aminoglycosides are typically administered via parenteral routes, except for cases involving intestinal infections or the need for decontamination. The protein binding is low, ranging from 0 to 30%. Additionally, the apparent elimination half-life is roughly 2 h. The primary method by which aminoglycosides are removed from the body is through the kidneys, with just a small portion excreted through the bile (0.5–2% of the amount administered). The biotransformation is minimal, accounting for less than 10%. These substances are predominantly present in their original, physiologically active state in the urine. The pharmacokinetics of elimination are not influenced by the dosage or method of administration [96].

**Table 4:** Clinically relevant DDIs for aminoglycosides.

Drug	Type	Mechanism	Refs.
ACEi, ARB	Pd	Increased risk of nephrotoxicity.	[97]
Antineoplastics	Pd	Both cisplatin and aminoglycosides induce sensorineural hearing loss. This is primarily caused by damage to the outer hair cells, specifically in the basal turn of the cochlea. Both classes of ototoxic medications induce oxidative stress within the inner ear, which serves as the primary catalyst for cellular damage. Additionally, there is an elevated risk of nephrotoxicity.	[98,99,100]
Biphosphonates	Pd	Additive effect that could lower calcium levels in the blood for a long time.	[101]
Diuretics	Pd	Increased risk of ototoxicity.	[99,100,102]
Immunosuppressants	Pd	Increased risk of nephrotoxicity.	[103]
Lincosamides	Pd	Increase in the neuromuscular blockade effect produced by aminoglycosides.	[104]
Muscle relaxants	Pd	Enhanced neuromuscular blockade, increasing the risk of respiratory depression and prolonged muscle paralysis.	[99]
NSAIDs	Pd	Increased aminoglycosides plasma concentrations, enhancing side effects.	[84,99]
Opioids	Pd	Increased risk of neuromuscular blockade.	[99]
Radiographic contrast agents	Pd	Increased nephrotoxicity.	[105]
Vancomycin	Pd	Increased risk of ototoxicity and nephrotoxicity.	[106]

Legend: Pk—pharmacokinetic; Pd—pharmacodynamic; ACEi—angiotensin-converting enzyme inhibitors; ARB—angiotensin receptor blockers; NSAIDs—non-steroidal anti-inflammatory drugs.

Aminoglycosides are frequently indicated for patients with severe sepsis as a complex anti-infective antibiotic regimen component [107]. This variant becomes an option when acquired infections are life-threatening, especially those caused by *Acinetobacter baumanii* (the emerging cause of nosocomial infections), *Pseudomonas aeruginosa*, and *Staphylococcus aureus*. In this category of drugs, service concentration determination is a pertinent surrogate for tissue concentration determination to assess antimicrobial dose and exposure adequately [108,109].

### 2.5. Tetracyclines

A large variety of bacteria, including Gram-positive and Gram-negative strains, spirochetes, intracellular bacteria, and protozoan parasites are susceptible to tetracycline antibiotics. The original tetracyclines were actinomycete fermentation byproducts [110].

With the exception of tetracycline, which is excreted as the metabolite Δ-epitetracycline, first-generation tetracyclines undergo metabolism, with just 5% being excreted in this form. These agents are eliminated unaltered through the renal and biliary pathways. The quantity of medication eliminated by urine is less than 50%. More than 40% of medications are excreted in the faeces through biliary clearance, and most tetracyclines undergo enterohepatic circulation. Biliary concentrations can surpass blood values by a factor of 5. Doxycycline undergoes minimal metabolism. Doxycycline is excreted without undergoing any changes in both the renal and biliary pathways [111]. The serum half-lives of the different substances are as follows: oxytetracycline, tetracycline, and demeclocycline have half-lives of 12 to 16 h; methacycline has a half-life of 14 to 16 h; minocycline has a half-life of 11 to 18 h; and doxycycline has a half-life of 15 to 25 h [112].

Eravacycline is a newly approved tetracycline derivative that might gain popularity in clinical practice due to its efficacy. It is an entirely synthetic compound that includes one fluorine atom as well as a pyrrolidineacetamide group on the side chain at the C9 position on its D-ring. This distinctive structure offers a defence against tetracycline-specific mechanisms of resistance employed by both Gram-positive and Gram-negative bacteria [113]. Table 5 illustrates the clinically relevant DDIs for tetracyclines class.

Eravacycline may be a suitable option for individuals who are at risk of developing *Clostridium difficile* infection. Additionally, eravacycline is effective in treating various types of infections caused by resistant Gram-negative bacteria and mixed infections. It is considered more tolerable and has a better safety profile compared to tigecycline, based on current research [118]. The simultaneous administration of eravacycline with both a CYP3A4 inducer and inhibitor shows a minimal likelihood of clinically relevant drug-drug interactions, especially when strong CYP3A inducers are involved. In such cases, a dosage adjustment is necessary to guarantee adequate exposure [119].

### 2.6. Other Classes

Vancomycin is used extensively in clinical practice to treat Gram-positive germs, including methicillin-resistant *Staphylococcus aureus* [120]. The protein binding of vancomycin is relatively low, typically reported to be around 50–55%. Vancomycin is excreted without undergoing any changes in the kidneys, and the dosage should be decreased in patients with impaired kidney function. Vancomycin exhibits a biphasic elimination half-life, characterised by a rapid initial half-life and a terminal half-life of 4 to 6 h in healthy people with normal kidney function. Patients with impaired kidney function have a notable increase in the time it takes for the drug to be eliminated from their body [121].

Vancomycin possesses bactericidal activity due to the ratio of the area under the curve to the minimum inhibitory concentration. Also, the starting dose is calculated based on special medical conditions, such as the presence or absence of renal insufficiency or based on the patient’s body mass [122,123]. Because of the pharmacotoxicologic profile with multiple implications of adverse reactions, the efficacy evaluation of vancomycin-initiated antibiotic therapy should be performed cautiously and with carefully evaluated drug–drug interactions (Table 6) [124,125]. Mainly, the most common complications of vancomycin monotherapy are attributed to renal impairment and those associated with vancomycin infusion; therapeutic monitoring is of significant importance in this case [126].

Linezolid undergoes largely hepatic metabolism, forming two metabolites: an amino ethoxy acetic acid metabolite and a hydroxyethyl glycine metabolite. These metabolites are produced as a result of oxidation of the morpholine ring. The more prevalent of the two metabolites, the hydroxyethyl glycine metabolite, is believed to be produced through non-enzymatic mechanisms. Although the exact enzymes involved in linezolid metabolising are unknown, it does not seem to undergo transformation through the CYP450 enzyme system. Additionally, it does not significantly inhibit or stimulate the activity of these enzymes. The predicted elimination half-life ranges from 5 to 7 h [137].

Linezolid is frequently contraindicated and associated with multiple significant DDIs [138]. Its use in combination with serotonergic drugs has been intensively studied because of the occurrence of severe serotonergic syndrome, a life-threatening complication. Among the medications incriminated to interact significantly with linezolid are antidepressants, vasopressors, opioids, and agents with dopaminergic mechanisms [17,139]. The mechanism postulated to be involved in this DDI is based on the connection between excessive serotonin accumulation in the central nervous system. Linezolid inhibits the enzyme monoamine oxidase in a non-selective manner and thus inhibits the metabolic processes of the monoamine neurotransmitter, which results in an abundance of serotonin in the central nervous system [140].

Dai et al. found that linezolid is responsible for more than three out of ten of the most common contraindicated DDIs [141]. In a multicentre study, linezolid was found to be the most frequently contraindicated drug in all instances of DDI. However, clinicians are unaware of the potential risks associated with this drug and often prescribe it as a therapeutic anti-infective agent to treat nosocomial infections [142].

Nevertheless, a recent meta-analysis showed that vancomycin and linezolid have a robust therapeutic efficacy against multiple nosocomial infections. The effectiveness of linezolid therapy was between 84.4% and 94%, and for vancomycin, between 76.9% and 90% [143]. For this reason, the prescription of these drugs is a valuable tool in global efforts to control nosocomial infections. Still, it requires particular caution because of the potential life-threatening DDIs in critically ill patients.

Polymyxins, types B and E, are used extensively in clinical practice [144]. The ever-increasing prevalence of pneumonia caused by multidrug-resistant Gram-negative bacteria, together with the decreasing availability of therapeutic options, is a worrying factor for health systems. Consequently, polymyxins, although a very old antibiotic, have started to be commonly used in the treatment of nosocomial infections with multidrug-resistant germs, as they show bactericidal activity against most aerobic Gram-negative bacilli [145,146,147].

Colistin does not undergo absorption when administered orally. After administering colistin sulfate through an intravenous bolus, just 0.18 ± 0.14% of the entire colistin dose is found in the urine after a 24-hour period; hence, colistin experiences significant reabsorption in the renal tubules by a mechanism facilitated by carriers, and its elimination primarily occurs through non-renal routes. The primary route of elimination for colistin is through the kidneys. Following parenteral injection, almost 60% of colistin methanesulfonate is eliminated in the urine within the initial 24 h [148].

In general, in the presence of renal impairment or concomitant administration of nephrotoxic drugs, the administration of polymyxins is contraindicated [149]. Additionally, if polymyxins are the only therapeutic option, monitoring fluid intake and determining electrolyte levels is necessary [150]. Moreover, polymyxins possess a transient pharmacotoxicologic profile, meaning that dizziness and tremor disappear with treatment discontinuation. However, at the onset of neurotoxicity symptoms, polymyxins and other co-administered neurotoxic drugs should be discontinued as soon as possible [151].

## 3. ADME Considerations for DDIs

### 3.1. Absorbtion

Changes in gastric pH might affect the solubility or chemical stability of many oral antimicrobials, particularly certain β-lactams. Proton-pump inhibitors or H2-receptor antagonist medications can alter the oral bioavailability of these agents. Cationic anti-acids, such as magnesium or aluminium, calcium and iron to a lesser extent, along with sucralfate and kaolin–pectin may generate insoluble compounds with tetracyclines, fluoroquinolones, and lincosamides. This type of interaction reduces the absorption [78].

A notable example is the pharmacokinetic profile of macrolides, which vary depending on their chemical composition. Erythromycin undergoes degradation in acidic environments. The 8,9-anhydro-6,9-hemiketal intermediate lacks antibiotic activity but can lead to gastrointestinal side effects similar to those observed with erythromycin. Subsequently, this intermediate undergoes additional metabolic processes to become the inert compound anhydroerythromycin, also known as erythromycin-6,9;9,12-spiroketal. Clarithromycin exhibits improved acid stability compared to erythromycin and undergoes less degradation in the stomach. Azithromycin exhibits a stronger stability at low pH levels, leading to a prolonged serum half-life and higher tissue concentrations in comparison to erythromycin. Azithromycin exhibits an oral bioavailability of 37%, whereas clarithromycin has an oral bioavailability of 55%; in contrast, erythromycin has a lower oral bioavailability of 25% [56,152]. Any pH modification can alter the absorption of these antimicrobials.

### 3.2. Distribution

Drug interactions are hypothesised to rapidly alter the binding of proteins, leading to changes in the concentration of free drugs. This is often mentioned as a potential cause of adverse drug responses. However, the rise in the concentration of unbound drugs is only temporary, as the processes of drug distribution and drug removal adjust to counterbalance it [153]. Drugs with a high level of binding to plasma proteins are more susceptible to displacement by drugs with a stronger attraction to the same attachment site. From a clinical perspective, removing a drug can potentially lead to side effects or toxicities if the replaced drug has a high affinity for plasma proteins (>90%), a smaller distribution volume, a narrow therapeutic range, and a rapid onset of action [154].

Around 35–40% of patients in the intensive care unit have a severe albumin deficiency, with serum albumin concentrations below 2 g/dL. This factor should be considered when treating patients with highly bound antibacterial agents (those with protein binding >80%), particularly since these antibiotics are eliminated through the kidneys to some extent. The decreased albumin concentration is anticipated to enhance the proportion of unbound drugs available for renal clearance, leading to suboptimal drug levels [99].

DDIs are often caused by the activation or inhibition of drug transporters, which are responsible for the movement of substances into and out of cells. The drug transporters found in the small intestine, liver, and kidney play an important role in determining the pharmacokinetic characteristics of pharmaceuticals. DDIs mediated by transporters can significantly impact the pharmacokinetics and therapeutic outcomes of the administered drugs [155]. Drug membrane transporters facilitate the entry of substances into phase I reactions and the subsequent removal following phase II reactions. Drug uptake transporters transfer the substance to an intracellular biochemical detoxification system, while drug efflux transporters reduce the amount of drug inside the detoxification system. Blocking transmembrane transporters can result in reduced absorption of the substance, leading to limited entry of the drug into the cells and decreased interaction with the enzymatic systems [156].

P-glycoprotein (P-gp), also known as multidrug resistance protein (MDR1), is a type of adenosine triphosphate (ATP)-coupling cassette transporter (*ABCB1*) that is extensively studied due to its role in limiting effective cancer pharmacotherapy. P-gp inhibits the cellular absorption of a wide range of both functional and structurally varied substances, including the majority of cancer treatments, hence leading to multidrug resistance [157]. P-gp has been extensively investigated primarily because of its ubiquitous expression. The induction of intestinal P-gp can enhance the presystemic evacuation of its substrates, leading to a decrease in their oral bioavailability. Conversely, inhibition can enhance the extent to which medications that are substrates of P-gp are absorbed into the bloodstream through oral administration [155].

P-gp appears to affect the distribution of many antibiotics. P-gp plays a role in the transportation of tobramycin, azithromycin, and clarithromycin in the gastrointestinal tract, and it has the ability to limit their absorption in the intestines. The absorption of macrolides in the intestine is believed to be restricted by efflux transporters P-glycoprotein [158]. Erythromycin and clarithromycin contribute to DDIs due to their involvement in the distribution of many other pharmacologic agents. For example, erythromycin and clarithromycin enhance the absorption and levels of pravastatin and simvastatin by delaying their elimination through P-gp [159]. P-gp was identified as an efflux transporter for minocycline and sparfloxacin at the blood–brain barrier. P-gp is also implicated in the excretion of fluoroquinolones in various compartments, including the gastrointestinal, renal, hepatic, and transepithelial compartments. This efflux transporter is especially significant in pharmacogenomics due to its role as a P-gp inhibitor for drugs like minocycline. Minocycline, for instance, has been discovered to raise the levels of riluzole in the blood plasma. Due to this effect, it is regarded as an antibiotic that possesses anti-neurodegenerative characteristics [26].

Organic anion-transporting polypeptides (OATPs), which are encoded by the *SCLO* genes, belong to the SLC family 21 and are responsible for facilitating the movement of a wide range of substances across cell membranes. OATPs are sodium-independent transporters located in the plasma membrane. They transport several substances derived from the body’s own metabolic processes [160]. Considering that OATPs play a role in the cellular uptake of multiple xenobiotics, their induction increases the concentration of OATP substrate drugs at the intracellular level, which may potentiate the effect and increase the risk of toxicity and adverse reactions. Conversely, OATP inhibition may lead to subtherapeutic concentrations of the administered drugs. Due to the vast range of substrate recognition of OATPs, multiple therapeutic agents, when taken together, may mutually affect each other’s pharmacokinetic profiles by interacting with the same transporters, either in a competitive or non-competitive manner, which causes DDIs [161]. β-lactams are substrates of OATP, which facilitate their absorption in the kidneys and intestines. Probenecid, a substance that inhibits the OAT system, prevents the kidneys’ elimination of penicillin, hence enhancing its effectiveness. Moreover, several cephalosporins have been found to strongly block human OATP1, OATP2, OATP3, and OATP4 [162]. Erythromycin and clarithromycin are transported up into hepatocytes by OATP1 and OATP3, acting as substrates for these transporters. Azithromycin, unlike erythromycin and clarithromycin, does not appear to have any interaction with OATP1 or OATP3. The *SLCO1B1* gene is responsible for transporting erythromycin into the liver. The SLCO1B1*5 (rs4149056) variant is known to cause a 50% decrease in erythromycin transport. This variant accounts for approximately 10% of the differences observed in erythromycin demethylation, as measured by the erythromycin breath test. In addition, genetic variation in the *SLCO1B3* gene has been linked to changes in the buildup of erythromycin in the liver. Specifically, a variant at the 334 locus (rs4149117) has been found to enhance transporter activity and increase the absorption of erythromycin [56,163].

Multidrug resistance proteins (MRPs) belong to the ATP binding cassette (ABC) efflux transporter family. They play an important role in controlling the effectiveness of a wide variety of antiretroviral and antituberculosis medications. MRPs also play a key role in removing drugs bound to glutathione and can control the level of oxidative stress in cells [164]. MRP2 and MRP4 appear to serve as pathways for removing some β-lactam antibiotics, such as cefazolin (only MRP4), from the human renal proximal tubule. The transport of ceftizoxime and cefazolin by MRP4 has been found to have affinities in the micromolar range. In addition, most cephalosporins that were examined showed a dose-dependent inhibition of MRP4-mediated transport; however, it is important to note that cephaloridine did not exhibit this inhibition [162]. Approximately 6% of azithromycin is found in the urine, whereas the majority is eliminated intact in the bile by MRP2 (encoded by the gene *ABCC2*) and P-gp (*ABCB1*). It is believed that MRP2 has a lesser impact on the elimination of azithromycin via the gall bladder than P-gp [165]. *ABCB1* and *ABCC2* genetic variations have been shown to influence the transportation and elimination of erythromycin. Patients who had the 2677GG (rs2032582) and 3435CC (rs1045642) diplotypes in the *ABCB1* gene exhibited higher maximal concentrations of azithromycin in comparison to patients with the 2677TT/3435TT diplotypes [56].

### 3.3. Metabolisation

The central mechanisms of clinically relevant DDIs involve the inhibition or induction of cytochrome P450 (CYP) enzymes. If a substance induces a CYP enzyme, it can enhance the biotransformation of a different medication or the compound itself (autoinduction), resulting in decreased levels in the bloodstream and an eventual reduction in the effectiveness of the drug. Conversely, evaluating the capacity of a substance to inhibit a particular CYP enzyme is extremely important as, when various pharmacologic agents are co-administered, they may impede each other’s metabolism, causing elevated toxicity [166,167]. Table 7 summarises the most important CYP enzymes related to antimicrobials.

Erythromycin is extensively metabolised by CYP3A4. Approximately 80% of the drug is inactivated through demethylation. Of the total amount, over 60% is eliminated from the body through the bile, and approximately 40% is expelled in the urine. The primary metabolite is N-desmethyl erythromycin. Clarithromycin is believed to undergo metabolism by CYP3A4, resulting in the production of two metabolites: N-desmethylclarithromycin, which is inert, and 14-(R)-hydroxyclarithromycin, which is active. Clarithromycin and erythromycin are believed to inhibit CYP3A4 by creating inactive compounds with CYP3A4 via their nitrosoalkane byproducts [168]. Additionally, erythromycin has been found to inhibit CYP3A4 and, also, P-gp. As a result, it causes a six-fold rise in the area under the curve of simvastatin via CYP3A4 inhibition. In addition, rhabdomyolysis is linked to the simultaneous administration of erythromycin and lovastatin, possibly because lovastatin concentrations rise due to decreased metabolism and limited efflux [169]. Nevertheless, studies have demonstrated that azithromycin has limited interaction with CYP3A4, as it is a modest substrate for the enzyme, undergoes minimal metabolism, and does not affect the activity of CYP3A4. In addition, ciprofloxacin is mainly metabolised down by CYP1A2, and is recognised as a known inhibitor of this enzyme [170].

### 3.4. Elimination

The elimination of pharmacologically active substances from the body can be influenced by several interactions, including excretion by an additional medication in the same organ. The kidney is the main organ responsible for the excretion of pharmaceuticals and their metabolites. DDIs at this level may arise due to competitive mechanisms in active tubular secretion when two or more drugs utilise the same transport route [171]. For example, it was shown that amoxicillin reduced the rate at which methotrexate is eliminated from the kidneys. Probenecid, a strong inhibitor of the renal tubular secretion anionic route, significantly enhances the area under the concentration–time curve of oseltamivir by 2.5 times [172]. Nevertheless, this competition among pharmacologic agents can be regulated for therapeutic intentions. Probenecid can enhance the levels of penicillins and cephalosporins in the blood, delaying their removal by the kidneys. This can result in a reduction in the required dosage [173]. Probenecid functions by competitively blocking OATP in the renal tubules. This action leads to an increase in the plasma concentrations of other substances that interact with the same transporter while decreasing their excretion [174].

Additionally, when urine pH is alkaline, the absorption of acidic medications declines, and in an environment with an acidic pH, the absorption of basic drugs decreases. However, the significance of variations in urine pH is only relevant if the pKa of the pharmacologic agent—which is the pH in which 50% of the molecules that exist in solution are in ionised form—falls within the range of 7.5 to 10.5 for bases and 3.0 to 7.5 for acids [172]. Antibiotics compatible with alkaline conditions include fluoroquinolones, aminoglycosides, and trimethoprim. Antibiotics compatible with acidic conditions include fosfomycin, tetracycline, nitrofurantoin, and some β-lactams. Based on their meta-analysis findings, Ordaz et al. proposed that conducting urine cultures with an antibiogram in both acidic and alkaline environments can be used to determine bacterial susceptibility characteristics. There is a lack of conclusive data from experiments conducted on living organisms to determine if selecting an antibiotic based on a patient’s urine pH or using substances to modify urinary pH will result in a better therapeutic outcome [175].

Comprehending the concepts of half-life is valuable for calculating the rate at which a particular drug is eliminated from the body and the stable concentration it reaches over time. The half-life of a substance is particularly relevant in cases when drug toxicity is a concern. These incidents can occur when patients are prescribed an incorrect dosage. It can also happen when patients have significant kidney or liver failure or when DDI can cause blood levels to exceed a toxic threshold [176]. The duration of a pharmacologic agent’s effectiveness and the probability of DDIs can be affected by its half-life. When two medications with disparate half-lives are co-administered, the medication with the shorter half-life undergoes more rapid elimination from the body compared to the medication with the longer half-life. Approximately 94 to 97% of the substance will be removed from the body after undergoing four to five half-lives. Therefore, it can be deduced that after four to five half-lives, the levels of a specific drug in the bloodstream will drop below a medically significant concentration, indicating that the drug has been removed [177]. It is important to consider that antibiotics with a short half-life have a narrow DDI onset window. Thus, analysing the information regarding the half-life is necessary to adjust the dosage of other co-administered drugs. However, in the case of drugs with long and very long half-times, where the DDI onset window extends over a long period, additional caution is required, and clinicians need to carefully assess the risk in order to make a decision that avoids DDIs.

## 4. Clinical Implications of DDIs in Nosocomial Infections

**Figure 3:**
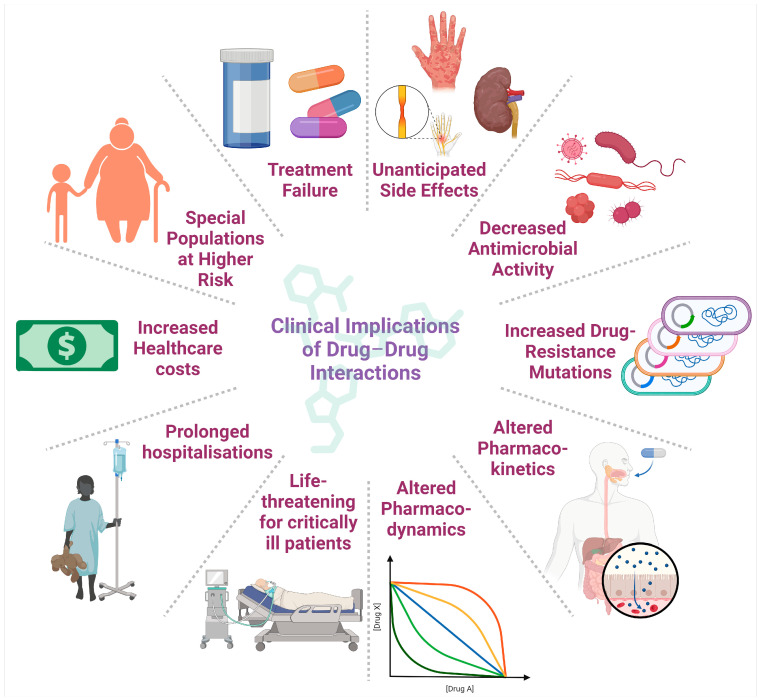
Overview of the clinical implications of drug–drug interactions in nosocomial settings (created with Biorender.com, accessed on 8 June 2024).

Nosocomial infections pose an imminent risk to patient safety and can occur in all sectors associated with health care. They can worsen chronic pathologies, cause death, and increase total hospitalisation costs. In practice, the incidence of these infections is influenced by a multitude of factors, such as the increasing proportion of the ageing population, the continuing emergence of antibiotic-resistant bacteria, and the complexity of medical treatments [178,179,180]. From a pharmacological point of view, DDIs occur when multiple drugs are administered simultaneously to treat one or more conditions and lead to unanticipated adverse reactions, life-threatening effects, or decreased therapeutic efficacy [96]. Figure 3 schematically illustrates the most important clinical implications of DDIs in nosocomial infections.

One of the main reasons for prescribing poly-drug regimens is to hinder the progression of antibiotic resistance [181]; thus, many separate mutations can occur that confer resistance to a mixture of drugs targeting different biological components. Drug combinations with antagonistic effects result in a slower onset of resistance compared to synergistic combinations [182]. For example, recent research on *S. aureus* has indicated that this pattern may not be universally applicable when bacteria develop increased levels of resistance, as the drug interactions themselves could be altered by resistance mutations [10,183].

The reduction in antimicrobial activity caused by DDIs is an important factor to consider. When providing concomitant administration of a drug with a CYP P450-inducing effect with another antibiotic necessary for the destruction of a microorganism, a marked decrease in the plasma concentration of the antibiotic occurs, hence, a reduction in antimicrobial activity [184,185]. This type of interaction is particularly dangerous, especially for critically ill patients who require the concomitant administration of various drugs [186].

For these reasons, antimicrobial stewardship programs are being optimised in current clinical practice. These are coordinated efforts for the rational use of antibiotics and involve administering the correct drug in the correct dose and with the most effective posology [187]. Essentially, these stewardship programs aim to improve patients’ health outcomes, reduce antimicrobial resistance, and lower healthcare costs [188].

DDIs mainly affect patients by reducing the efficacy of treatment and increasing the length of hospitalisation, thus increasing the costs associated with hospitalisation [189,190]. In a recent study by Schmitt et al., the authors concluded that the incidence of DDIs is increased in patients with community-acquired pneumonia. This was hypothesised to occur both because of the increased number of DDIs occurring during hospitalisations and the severity of these interactions. Furthermore, the presence of DDIs is a separate factor affecting the duration of hospitalisation [191]. In a prospective cohort study, Laurent et al. found that patients with multiple comorbidities are at higher risk of becoming infected with a nosocomial microbe. The most frequent diseases predisposing to nosocomial infections were respiratory pathologies and renal impairment [192]. Additionally, an important thing to mention is that patients with chronic conditions present a substantial challenge in evaluating the pharmacokinetic and pharmacodynamic profile of drugs [193]. The treatment may have either increased or decreased efficacy in this patient population, resulting in therapeutic failure and reduced treatment efficacy. This may be explained by the fact that most information on drug pharmacokinetics is obtained from healthy individuals and does not consider the complex interrelationships at different stages of organ failure or with the worsening of pathologies [194]. Moreover, studies performed on critically ill patients cannot provide conclusive information because of the unique characteristics of this population, the small number of studies, and the wide variety of drugs and interventions that can be applied in these cases [195].

## 5. Clinical Management of DDIs in Nosocomial Infections

**Figure 4:**
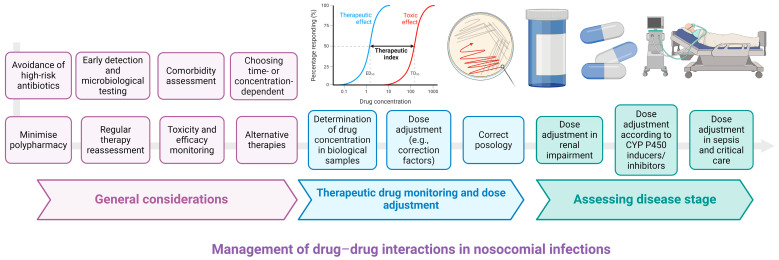
Strategies to manage DDIs in healthcare-associated infections (created with Biorender.com, accessed on 8 June 2024).

Identifying the most significant and medically relevant DDIs in primary care is of utmost importance to patient safety. Methods for mitigating the likelihood of DDIs involve reducing the number of prescribed medications, regularly reassessing therapy, exploring nonpharmacologic alternatives, vigilantly monitoring for indications of toxicity or efficacy, modifying medication dosages as necessary, and adapting administration schedules [196]. A general rule for the management of DDIs in nosocomial infections is the use of alternative therapies whenever possible [197]. The patient’s associated comorbidities should be assessed before initiating antibiotic therapy. This involves creating an individualised antibiotic regimen based on evaluating the risk of DDIs between antibiotics and the other drugs used by the patient [198]. An example would be situations of serious drug–drug interactions, such as those triggered by linezolid, vancomycin, imipenem, etc., in which the patient’s life is jeopardised. In these situations, antibiotics that may cause problems associated with high-severity adverse events should be avoided, and safer therapeutic alternatives should be found. Figure 4 illustrates the most important strategies for managing DDIs in nosocomial settings.

Another critical factor to consider is the accurate assessment of time-dependent antibiotics, i.e., concentration-dependent antibiotics. Under the first category are the β-lactam or vancomycin antibiotics, which have a time-dependent bactericidal effect and are only marginally influenced by the concentration of the drug above the minimum inhibitory concentration (MIC). The bactericidal effect of these drugs is generally gradual, and increasing the concentration above a threshold of peak killing activity—usually four times the MIC—does not significantly increase the bactericidal activity [49,199]. Generally, when administering time-dependent antibiotics, it is necessary to minimise the time the drug concentration remains above the MIC. On the other hand, when administering concentration-dependent antibiotics, the ratio of the peak drug concentration to the MIC is the essential pharmacodynamic aspect required for optimising therapy [200]. This category comprises drugs demonstrating concentration-dependent bactericidal activity and marked post-antibiotic effects. In this category are antibiotics such as aminoglycosides, azithromycin, colistin, metronidazole, and fluoroquinolone. The efficacy of concentration-dependent antibiotics is determined by the time-dependent killing rate and the retention time of the post-antibiotic effect. This means that the amount of drug, based on the maximum concentration and the area under the concentration–time curve relative to the minimum inhibitory concentration, is more important than the frequency of dosage in determining their efficacy [199,201].

Therefore, understanding the pharmacokinetic characteristics of antibiotics can assist clinicians in determining whether to reduce the dose and maintain the frequency of administration (typically recommended for time-dependent antibiotics) or keep the dose unchanged and extend the interval between doses (typically recommended for concentration-dependent antibiotics) when adjusting the dosage for patients with kidney disease [200].

### 5.1. Therapeutic Drug Monitoring and Dose Adjustment

Therapeutic drug monitoring (TDM) represents the assessment of drug concentrations in plasma, blood, or other biological samples; the ultimate goal is to determine the most effective dosage for the patient. TDM is of significant importance because a patient may receive multiple drugs to treat different conditions, and these drugs may interact and cause adverse effects [202].

Pharmacokinetic DDIs for different antibiotic classes can be efficiently assessed by determining antibiotic concentrations [99,199]; however, these types of DDIs are complex and require analytical techniques capable of evaluating a wide variety of active molecules. These methods would need to be introduced into clinical practice rapidly and with high specificity in order to accurately assess the effect of antibiotics on drugs with very narrow therapeutic indexes, such as antifungals, antiretrovirals, immunosuppressants, antiepileptics, or antipsychotics [99,203].

An important example in clinical practice is the interaction between macrolides and antipsychotics. In general, when a patient chronically treated with antipsychotics acquires a macrolide-sensitive strain, it is necessary to re-evaluate the case and find other antibiotic therapy options. If this is not possible, then TDM should be performed. If TDM assessment methods are not available, a correction factor equal to 0.50 can be applied to the antipsychotic dose. This is calculated based on the effects that CYP3A4 inhibitors have on antipsychotics [60,204]. The same reasoning applies to the combination of fluoroquinolones and antipsychotics, but in this case, the correction factor is equal to 0.33. Also, QTc monitoring is mandatory [60].

Erythromycin and clarithromycin increase the absorption of cyclosporine by inhibiting its metabolisation in the intestinal wall [71]. If their combination is necessary and no other option is available, a decrease in the cyclosporine dose by 35–50% is required. Additionally, frequent daily monitoring of calcineurin inhibitors and up to 72-hour monitoring of mTOR inhibitors is necessary [69].

Oral coumarins are the most commonly prescribed anticoagulants, as they are cost-effective and widely available [205]. Active substances in this class have a very narrow therapeutic index that requires frequent monitoring and dose adjustment. Patients on long-term treatment experience a higher international normalised ratio (INR) when given antibiotics that inhibit cytochrome-P450. As a result, these patients need a lower dosage of warfarin in order to reduce the likelihood of severe bleeding. Combining warfarin with antibiotics that stimulate cytochrome-P450 has been demonstrated to reduce INR levels, necessitating higher doses to obtain the desired anticoagulant effect. Additional variables that can impact the dosage of warfarin in people taking antibiotics encompass age and infection conditions [40].

### 5.2. Management of DDIs in Kidney and Liver Impairment

DDIs may cause increased toxicity due to combined pharmacokinetic or pharmacodynamic effects. The introduction into the therapy of new active substances or repurposing already existing molecules in the treatment of critically ill patients may result in an increase in renal toxicities, which are typically difficult to anticipate or identify [206].

Important considerations include interventions performed to improve the selection and administration of anti-infective agents in renal failure. Dose adjustment according to renal clearance is frequently overlooked [207]. Non-adherence to guidelines in hospitalised patients occurs between 19% and 67%. Available studies indicate that, in practice, recording glomerular filtration rate values has no impact on prescribing practices for nephrotoxic drugs, such as certain classes of antibiotics [208,209]. Existing creatinine-based equations for assessing renal function are based on understanding steady-state conditions. Consequently, these equations are inadequate for accurately estimating the glomerular filtration rate in acute kidney injury. The equations developed for managing severe renal failure are based on multiple serum creatinine measurements and utilise the principles of mass creatinine balance; however, these equations are based on the assumption of constant creatinine production rates and volume of distribution, which may not be accurate, especially in hemodynamically unstable patients [210]. The most commonly prescribed antimicrobials that require dose modification in renal failure are vancomycin, colistin, meropenem, piperacillin/tazobactam, ciprofloxacin, and fluconazole [211].

At the hepatic level, the most common and dangerous DDIs are the inhibition or activation of enzymes that metabolise drugs. These interactions lead to premature discontinuation or withdrawal of certain medications from clinical practice. The management of inhibitory or inducible DDIs is challenging to achieve as there is a large inter-individual variability in terms of intensity [186,212]. In patients with severe liver failure, reversible liver enzyme inhibition is extremely low or almost completely disappears. This reduction is caused by decreased hepatic absorption of inhibitory drugs or decreased enzyme expression. However, irreversible interactions may be only partially reduced, as they are exclusively affected by the reduced expression of the enzyme that has been inhibited [213].

A particular category of patients is represented by those with viral hepatitis, who are given multidrug regimens and who are at increased risk of DDIs [214]. Additionally, they may acquire a nosocomial infection once hospitalised due to low immunity [215]. Noor et al. have demonstrated that patients with viral hepatitis have a large number of significant DDIs, and their evaluation is particularly important for the success of therapy and increased life expectancy in this category of patients [216]. In patients with cirrhosis, the assessment and management of DDIs is particularly difficult to quantify. In a recent meta-analysis, Dafonte et al. highlighted that the assessment of the correct dose for patients with liver cirrhosis is conditioned by various patient-related factors, including increased body fluid levels, disturbed electrolyte balance, reduced serum albumin and other protein levels, decreased muscle mass, presence of portosystemic shunts, and impaired hepatic metabolism with a diminished first-pass effect. In addition, drug-related factors such as hepatic extraction or bioavailability, metabolic pathways, protein binding, and route of administration also play a role in dose calculation [217].

In general, antibiotics are widely recognised as drugs that can induce liver damage. While the occurrence of severe antibiotic-induced liver damage is relatively rare compared to the large number of prescriptions given each year, population-based estimates indicate that it happens in less than 5 out of every 100,000 people. However, it remains a significant cause for discontinuing antibiotics once they are released to the market. Antibiotic-induced hepatotoxicity typically does not show any symptoms, is temporary, and is linked to only minor liver damage. Occasionally, there are instances where there is a notable level of illness, the requirement for a liver transplant, and even death resulting from sudden liver failure [218,219]. Amoxicillin–clavulanate is the combination most frequently associated with drug-induced liver injury. Antibiotics directly impact the composition and variety of microorganisms in the gastrointestinal tract, as well as the alterations in metabolites. The reduction of probiotics following antibiotic intervention can diminish the effectiveness of hepatoprotective drugs, resulting in the manifestation of liver injury [220]. Multiple studies have shown that the decreased presence of genetically modified species and genes is the leading cause of individual vulnerability to drug-induced liver injury [221,222,223].

### 5.3. Assessing Disease Stage for Proper DDI Management in Nosocomial Infections

In general, to acquire a nosocomial infection, two specific conditions are necessary. These are represented by the lowering of the defence mechanisms of the respective host by the presence of pathogenic and non-pathogenic microorganisms [224].

In clinical practice, polytherapy is frequently used for the management of chronic pathologies [225]. For example, patients with HIV/AIDS—characterised by severe immunodeficiency and often requiring long-term hospitalisations—often require the use of combination pharmacotherapy in order to decrease the risk of resistance to antiretroviral therapy [226]. The same approach is required for patients with tuberculosis, malaria, and other infectious diseases that lower the body’s defence mechanisms. In such situations, monitoring DDIs is crucial, and unrecognised adverse reactions and therapy failure are almost inevitable in the absence of proper monitoring adapted to the severity of the pathology [227].

Moreover, the available medication must be carefully evaluated to avoid potentially harmful effects in treating critically ill patients. These potentially life-threatening adverse reactions may be interactions between several drugs administered concomitantly, drug–drug interactions with pathology, or incorrect dosing. All of these will result in a longer duration of illness, a denied quality of life, increased hospitalisation costs, and even death [228]. Understanding the pharmacodynamic characteristics of antibiotics and the pharmacokinetic changes in critically ill patients can help clinicians to tailor individualised dosing programs for each patient. Hydrophilic antibiotics are influenced by the pathophysiologic changes occurring in these groups of critically ill patients. These changes are represented by the increased volume of distribution and alterations in drug clearance from the body [229]. This class includes aminoglycosides, colistin, glycopeptides, and β-lactams [230]. Lipophilic antibiotics, on the other hand, may undergo alterations in the clearance rates from the body and the presence of minimal variations in the volume of distribution [229]. This class includes fluoroquinolones, lincosamides, some tetracyclines, and macrolides [231].

In these cases, the antibiotic loading dose can be determined by dividing the volume of distribution by the desired plasma concentration. Since renal function does not influence this reasoning, it is not necessary to evaluate creatinine clearance [232].

Patients presenting with sepsis have an increased volume of distribution for hydrophilic antibiotics, which leads to the need to increase the loading dose [233]. Additionally, the concentration of lipophilic antibiotics that can penetrate deep into adipose tissues is much less evident in the extravascular tissues [234].

Most nosocomial infections originate from the patient’s own bacterial flora, but the major problem is that critically ill patients become colonised with antibiotic-resistant strains. Unfortunately, a nosocomial infection has a significant influence on disease and mortality rates and an extremely high impact on increased hospitalisation and costs [224].

The identification of different strategies for disease management is a cornerstone for increasing the outcomes of therapies and improving the quality of life of all patient populations. The significance of early detection is a topic gaining increasing attention in the healthcare sector [235]. Microbiological techniques for expedited diagnosis enable prompt identification of bacteria, which is crucial for the timely treatment of patients [236]. The treatment of bacterial nosocomial infections should include isolating the bacteria in culture and conducting tests to determine their susceptibility to antimicrobial agents [237].

## 6. Key Findings and General Considerations

The majority of patients who are admitted to the hospital are prescribed many medications to manage their underlying health conditions, prevent infections acquired during their hospital stay, or treat nosocomial infections. This requires a comprehensive evaluation of DDIs, not only to prevent potential harmful effects but also to achieve optimal efficacy in pharmacological therapy [238]. However, despite the increasing number of interventions implemented to reduce the risk of DDIs, the prevalence remains high. A recent systematic review and meta-analysis found that the occurrence of clinically significant DDIs varied between 1.2% and 64.0% [189]. A different meta-analysis, which examined the frequency of DDIs in hospitalised patients, found that the combined prevalence of possible DDIs was 64.9%, while the combined prevalence of clinically significant DDIs was 17.17% [239]. Moreover, Wannawichate et al. highlighted that the rising prevalence of negative outcomes linked to DDIs is a frequent reason for hospitalisation, particularly among the elderly population [240].

Reis et al. conducted a cross-sectional retrospective analysis, which revealed a 70% prevalence of probable DDIs in the intensive care unit during specific periods of hospitalisation. The majority of the DDIs were classified into two categories: severe or moderate. Pharmacodynamic interactions exhibited a slight prevalence compared to pharmacokinetic interactions. The presence of possible drug interactions was linked to the quantity of drugs given and the duration of hospitalisation. Pharmacotherapy factors related to DDIs included inductors of cytochrome P450, medication that prolonged the QT interval, and cardiovascular drugs. The study identified an elevated risk of potential drug interactions in the intensive care unit as a result of the polypharmacy delivered. The interactions were correlated with the quantity of drugs, the duration of hospitalisation, and the properties of the prescribed medications [241].

**Figure 5:**
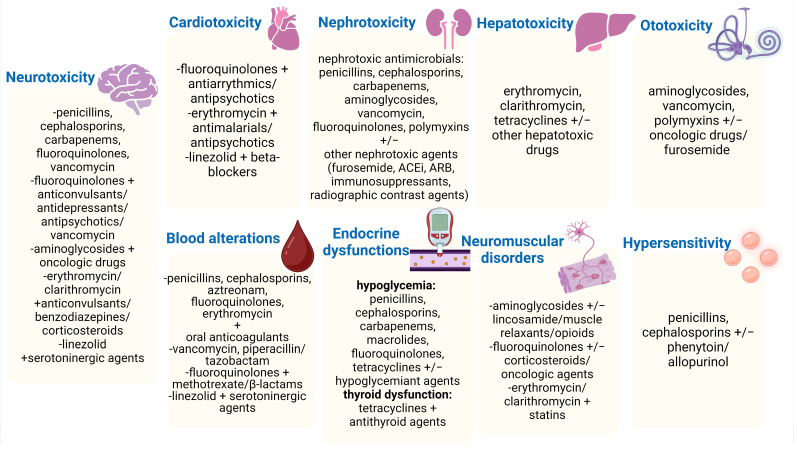
Categorisation of major DDIs and disease–antibiotic interactions for nosocomial infections. Legend: ACEi: angiotensin-converting enzyme inhibitors; ARB: angiotensin receptor blockers.

In consequence, data suggest that DDIs remain a major health risk for hospitalised patients. There is an urgent need to consider all the tools for improving the therapeutic outcomes of hospitalised patients, especially for those who acquire a nosocomial infection. Therefore, Figure 5 summarises the major DDIs in nosocomial settings, along with disease–antibiotic interactions by the type of health issue they provoke.

Predicting DDIs accurately can be challenging due to various confounding factors: patient characteristics (age and gender), physiological changes caused by drugs (changes in liver blood flow and protein binding), pathological changes induced by the intensive care unit treatment (low albumin levels and increased renal clearance), disease stage, polypharmacy, multiple comorbidities, infections with drug-resistant hospital germs, and complex dialytic procedures. All these factors can significantly impact the distribution, metabolism, and elimination of antibiotics, ultimately affecting the clinical significance of DDIs. An effective strategy for managing DDIs could involve implementing a “fast-track” clinical pharmacology approach at the patient’s bedside. This approach would involve using specialised software, checkers for drug interactions, and TDM of anti-infective and non-anti-infective medications whenever possible. There is now preliminary yet consistent evidence indicating that evaluating DDIs by clinical pharmacy specialists and continuously monitoring patients is a viable technique for optimising pharmacotherapy [99].

## 7. Conclusions

This narrative review evaluated clinically significant and relevant DDIs in nosocomial settings, highlighting their impact on patient safety and treatment failure. The high prevalence and the particularities of these DDIs require a robust method of prediction and management, especially in the case of antibiotic-resistant nosocomial strains.

Personalised therapy is gaining increasing importance as this approach emphasises the need for individualised treatment depending on the patient, the disease severity, and comorbidities. It considers the drug’s pharmacokinetic and pharmacodynamic parameters directly related to the patient’s characteristics. This strategy has the potential to maximise the efficacy of the treatment and reduce the likelihood of adverse reactions, improving overall patient outcomes. Clinicians need to engage in ongoing training to be able to prevent or easily identify DDIs.

An urgent need is to develop more efficient management strategies for DDIs in nosocomial infections. These should include high-frequency monitoring of therapeutic drug levels, consistent assessment of treatment efficacy, and consideration of non-pharmacologic alternatives where possible. Practice guidelines should emphasise the significance of individualised dosing regimens, especially in patients with kidney, liver, or critically ill conditions.

All these measures require coordinated efforts and modern prognostic and diagnostic technologies capable of providing real-time data. A future perspective may be implementing electronic patient histories linked to a decision guidance system to help physicians and pharmacists evaluate drug therapy and DDIs.

## Figures and Tables

**Table 1:** Clinically relevant DDIs in β-lactam class.

β-Lactams	Drug	Type	Mechanism	Ref.
Cef	Anticoagulants	Pd	Warfarin interacts with Cef that contain an NMTT side chain by inhibiting the production of blood coagulation factors. These effects are also exacerbated by Cef that lack the NMTT side chain.	[33]
Cef	Antiretroviral drugs	Pk	Antiretrovirals disrupt the functioning of P-glycoprotein (which actively moves drugs over cellular barriers). Co-administration impacts antiretrovirals’ absorption, distribution, and excretion, resulting in changes to their effectiveness and safety.	[34]
Cef	Calcium i.v. solutions	in vitro	Precipitation events that could harm vital organs.	[35]
Cef	Antidiabetic drugs	Pd	Cefditoren includes pivalic acid, which reduces the carnitine content in the bloodstream. This results in the inability to synthesise glucose, causing hypoglycaemia.	[36]
Cef	Phenytoin	Pd	Stevens–Johnson syndrome, caused by phenytoin, worsened due to cefepime.	[37]
Cef	Proton-pump Inhibitors	Pd	A higher likelihood of developing acute renal damage.	[38]
Pen	Antiepileptics	Pk	Impact on the plasma concentration of valproic acid, causing a considerable reduction and insufficient management of seizures.	[39]
Pen	Warfarin	Pd	An increase in INR levels and a risk of bleeding. This can occur anywhere between seven days after starting amoxicillin treatment and nine days after stopping it.	[40]
Pen + Cef	Allopurinol	Pd	Typical drug-induced widespread rashes.	[41]
Pen + Cef	Aminoglycosides	Pd	The β-lactam ring gradually reacts towards the amino group, forming a penicillin–aminoglycoside complex with decreased antibacterial activity.	[42]
Pen + Cef	Anti-acids	Pk	Out of the 13 β-lactams that were studied, 4 of them showed a significant decrease in bioavailability when taken with antacids or mineral supplements. The β-lactam with the most critical negative impact was cefdinir, whereas cefpodoxime proxetil showed a mild decrease in bioavailability when taken with antacids.	[43]
Pen + Cef	Diuretics	Pk	Furosemide inhibits organic ion transporters 1 and 3 (which play an important role in the active secretion of antibiotics such as β-lactams). The channel’s disturbance of the usual electrochemical gradient-expelled cations like Ca^2+^ and Mg^2+^, results in a significantly positive charge in the lumen. This might result in the paracellular reabsorption of antibiotics with a positive charge.	[44]
Pen + Cef	Methotrexate	Pk	Most β-lactams are low-pH organic acids that interfere with the renal tubular discharge of methotrexate and its byproducts, resulting in a decreased clearance.	[45]
Pen + Cef	Probenecid	Pk	Probenecid hinders the elimination of β-lactams via the renal tubules. This slows down their clearance and leads to higher levels of antibiotics in the bloodstream. Organic anion transporters are competitively inhibited by probenecid.	[46]
Carb	Valproic acid	Pk	Co-administration leads to a notable decrease in the plasma levels of valproic acid. The mean plasma concentration of valproic acid was 68.7 µg/mL; however, due to the interaction, it reduced to a mean value of 15.8 µg/mL, suggesting a reduction of 77%. This decline results in inadequate seizure management and other unfavourable consequences.	[39]
Azt	Oral anticoagulants	Pd	Increased risk of bleeding.	[40]
Azt	Furosemide	Pk	A moderate increase in the amount of aztreonam in the body.	[15]
Azt	Probenecid	Pk	A moderate increase in the amount of aztreonam in the body.	[15]
Azt	Entecavir, crizotinib	Pk	Both are transported by organic cation transporter 2. Co-administration leads to higher levels of the drugs in the bloodstream as they compete for transporters in the renal tubules.	[47]

Legend: Pk—pharmacokinetic; Pd—pharmacodynamic; Cef—cephalosporin; Pen—penicillin; INR—international normalised ratio; NMTT—N-methyl-thio-tetrazole; Carb—carbapenem; Azt—aztreonam.

**Table 2:** Clinically relevant DDIs for macrolides.

Drug	Type	Mechanism	Refs.
Anti-arrhythmics	Pk	After five days of macrolide treatment, there was a 50% reduction in the overall elimination of quinidine.	[58]
Anticoagulants	Pd	Increased likelihood of bleeding, with an odds ratio of 1.86. The average INR level rises from 2.7 to 3.6 when juvenile cardiac patients are given this combination.	[40]
Antiepileptics	Pk	An increased plasma concentration of carbamazepine (129%), which causes carbamazepine toxicity (including drowsiness, dizziness, ataxia, heart block, and liver failure). Also, a notable reduction in the plasma levels of pregabalin (a reduction of 17% in the AUC and a decrease of 13% in the Cmax).	[39]
Antimalarials	Pd	Prolongation of QT and cardiac adverse effects.	[59]
Antipsychotics	Pd	Macrolides strongly impede the effects of quetiapine and can cause severe adverse drug reactions. Macrolides increase the QTc interval by blocking the potassium channels in the heart.	[60]
Antiretrovirals	Pk	Clarithromycin interacts with NNRTIs and PIs, reducing their AUC and increasing the risk of toxicity, necessitating dose adjustments or monitoring. Azithromycin is preferred when available.	[61,62]
Benzodiazepines	Pk	Elimination of triazolam was decreased by 52%, leading to impaired psychomotor function and memory loss. The concurrent use of midazolam and erythromycin leads to a more than four-fold increase in the AUC and a 54% decrease in clearance for midazolam.	[63]
Calcium channel blockers	Pd	A decrease in blood pressure due to the widening of blood vessels, a higher likelihood of hospitalisation due to low blood pressure or sudden kidney damage.	[64]
Corticosteroids	Pk	Clarithromycin decreases the activity of CYP3A by 75%, potentially elevating the amount of prednisone in the bloodstream. Caution should be applied to prednisone-induced mania, even at extremely low doses of prednisone, in older individuals who have taken a CYP3A inhibitor.	[65]
Cyclosporine	Pk	Increased blood concentrations for cyclosporine.	[66]
Digoxin	Pk	Increased digoxin toxicity via inhibiting P-glycoprotein.	[67]
Hypoglycemiants	Pk	Hypoglycemia can be worsened by the presence of hepatic cytochrome P450 inhibitors. Approximately 12.3% of hypoglycemia incidents in patients who are taking sulfonylureas are believed to be linked to the use of antimicrobial medications, particularly fluoroquinolones, macrolides, sulfamethoxazole–trimethoprim, and azoles.	[68]
Immunosuppressants	Pk	Co-administration of clarithromycin and erythromycin with calcineurin inhibitors or mTOR inhibitors leads to a substantial rise in the immunosuppressant’s AUC and Cmax, increasing it by 3 to 10 times.	[69]
Sildenafil	Pk	The administration of clarithromycin resulted in a 1.86-fold rise in sildenafil’s Cmax and a 2.29-fold increase in the area under the AUC.	[70]
Statins	Pk	Co-administration results in higher levels of statins in the bloodstream, which increases the likelihood of experiencing statin-related side effects, such as myopathy and rhabdomyolysis.	[71]

Legend: Pk—pharmacokinetic; Pd—pharmacodynamic; INR—international normalised ratio; AUC—area under the curve; Cmax—maximum concentration; NNRTIs—non-nucleoside reverse-transcriptase inhibitors; PIs—protease inhibitors; mTOR—mammalian target of rapamycin.

**Table 3:** Clinically relevant DDIs for the fluoroquinolone class.

Drug	Type	Mechanism	Refs.
Anti-acids/Sucralfate	Pk-Pd	Formation of insoluble complexes, with decreased absorption and antimicrobial activity.	[77,78]
Anti-arrhythmics	Pd	QTc prolongation and cardiotoxicity.	[79]
Antidepressants	Pd	Increased risk of delirium, hallucinations, psychomotor agitation, paranoid delusions, and suicidal thoughts/attempts.	[80]
Antiepileptics	Pd	In vulnerable patients, the presence of GABA-like structures at seven sites in norfloxacin and ciprofloxacin can antagonise GABA receptors, further increasing the likelihood of having seizures. Fluoroquinolones also stimulate the NMDA receptor, lowering susceptibility to seizures.	[81]
Antipsychotics	Pd	Suppression of the heart’s potassium channels through additive inhibition.	[60]
β-lactams	Pd	Increased risk of thrombocytosis.	[82]
Clozapine	Pd	Increased concentration of clozapine, sedation, rhabdomyolysis, and increased risk of QT prolongation.	[78]
Corticosteroids	Pd	Co-administration can lead to a heightened risk of tendinopathy or tendon rupture, particularly in females, individuals over the age of 60, and those who have been taking fluoroquinolones for an extended period. Exercise caution when administering corticosteroid therapy to individuals with multidrug-resistant tuberculosis who are also taking fluoroquinolones.	[83,84]
Cyclosporine	Pk	Ciprofloxacin can enhance the toxicity of cyclosporine. Patients taking cyclosporine for an extended period should not use ciprofloxacin and should instead choose another antibiotic.	[85]
Digoxin	Pk	Increased risk of digoxin toxicity via increasing digoxin serum levels.	[86]
Immunosuppressants	Pk	Ciprofloxacin reduces mycophenolate mofetil levels by disrupting enterohepatic circulation and absorption. Drug level monitoring is recommended even though levofloxacin does not elevate the level of cyclosporine.	[69]
Hypoglicaemiants	Pd	Levofloxacin’s risk for hypoglycaemia was 5.13 times higher than that of cephalosporins; however, it was 9.40 times higher than that of penicillin antibiotics. Furthermore, the findings indicated that levofloxacin posed the most significant risk for hypoglycaemia, followed by moxifloxacin and ciprofloxacin. In addition, patients who were taking levofloxacin at the same time as insulin or sulfonylurea were more likely to develop hypoglycaemia.	[87]
Levothyroxine	Pk	The decrease in T4 AUC following the simultaneous use of ciprofloxacin and L-T4 is in line with suppressing a T4 uptake transporter in the intestines, maybe belonging to the OATP family. The idea suggests that the thyroid hormone transporters MCT8, MCT10, or LAT1/2, found in the intestine, could potentially be where the interaction between L-T4 and ciprofloxacin occurs.	[88]
Methadone	Pd	Increased methadone toxicity and a life-threatening interaction.	[89]
Methotrexate	Pk	Co-administration leads to higher blood levels of methotrexate, which raises the likelihood of developing anaemia and bone marrow suppression and increases the susceptibility to infections.	[78]
NSAIDs	Pd	Co-administration reduces the antibacterial effect.	[90]
Probenecid	Pk	Probenecid hinders the removal of fluoroquinolones from the kidneys by blocking their release through competitive inhibition of renal organic ion transporters. It is an inhibitor of the renal tubular anion secretion pathway, specifically targeting OATP 1 and 3. Probenecid caused a 16% rise in the AUC of ofloxacin and a 75% increase in ciprofloxacin in healthy volunteers.	[78,91]
Theophylline	Pk	The daily administration of 1000 mg of ciprofloxacin decreases the clearance of theophylline by 19–32%. Co-administration to elderly individuals was linked to a roughly two-fold rise in the likelihood of being hospitalised due to theophylline toxicity.	[78]
Warfarin	Pd	Increased risk of bleeding.	[78]

Legend: Pk—pharmacokinetic; Pd—pharmacodynamic; GABA—gamma-aminobutyric acid; AUC—area under the curve; Cmax—maximum concentration; NMDA—N-methyl-D-aspartate; T4—thyroxine; OATP—organic anion transporting polypeptide; MCT—monocarboxylate transporter; LAT—L-type amino acid transporter.

**Table 5:** Clinically relevant DDIs for tetracyclines.

Drug	Type	Mechanism	Refs.
Antacids	Pk-Pd	Chelating DDI that reduces the oral bioavailability by 80%.	[99,114]
Antifungals	Pk	When eravacycline is administered with a potent inducer of CYP3A, like rifampin, the dosage should be increased. Concomitant administering of rifampin resulted in a decrease of roughly 33% in total eravacycline exposure and an increase of approximately 50% in clearance. However, it is uncertain whether a dose adjustment with an inhibitor like itraconazole is necessary.	[99]
Antipsychotics	Pk	Increased plasma concentration for clozapine.	[60]
Antithyroid drugs	Pd	Non-immune-mediated thyroid dysfunction.	[115]
Cyclosporine	Pk	Administration of cyclosporine enhances the levels of orally ingested tetracyclines in the bloodstream. The findings strongly indicate that efflux pumps in the intestinal epithelium regulate the absorption of tetracycline from the gastrointestinal tract, even though cyclosporine further hinders renal and hepatic clearance.	[116]
Iron supplements	Pk	Reduced bioavailability of tetracyclines.	[117]
Oral contraceptives	Pk	Tetracyclines can decrease the effectiveness of oral contraceptives.	[99]
PPIs	Pk	PPIs may decrease the absorption of tetracyclines.	[114]

Legend: Pk—pharmacokinetic; Pd—pharmacodynamic; DDI—drug–drug interactions; PPIs—proton-pump inhibitors.

**Table 6:** Most frequent DDIs for vancomycin.

Drug	Type	Mechanism	Refs.
Aminoglycosides	Pd	Nephrotoxicity, ototoxicity.	[127]
Amphotericin B	Pd	Nephrotoxicity, ototoxicity.	[128]
Anticancer drugs	Pd	Nephrotoxicity, ototoxicity.	[129]
Antivirals for *H. simplex*	Pd	Neurotoxicity, nephrotoxicity.	[130,131]
Calcineurin inhibitors	Pd	Synergistic or additive renal impairment potential.	[132]
Cephalosporins	Pd	Neurotoxicity, nephrotoxicity.	[129,133]
Diuretics	Pd	Nephrotoxicity, ototoxicity.	[129]
Neuromuscular blocking agent	Pd	Increased risk of neuromuscular blockade.	[134]
NSAIDs	Pd	Nephrotoxicity.	[129,135]
Opioids	Pd	The combination of morphine with vancomycin significantly increases the amount of histamine released.	[136]
Polymyxins	Pd	Nephrotoxicity, ototoxicity.	[129]

Legend: Pk—pharmacokinetic; Pd—pharmacodynamic; NSAIDs—non-steroidal anti-inflammatory drug.

**Table 7:** Antimicrobials commonly used in nosocomial infections and CYP enzymes (adapted from [78,166]).

**CYP3A4**		
**Substrates**	**Inducers**	**Inhibitors**
opioid analgesics, alpha-1 adrenergic blockers, benzodiazepines, anti-arrhythmics, calcium channel blockers, anticoagulants, antimalarials, statins, antituberculars, tyrosine kinase inhibitors, corticosteroids, anxiolytics, anticonvulsants, macrolide antibiotics, lincosamide antibiotics, atypical antipsychotics, immunosuppressants (calcineurin inhibitors), antileprosy agents, antiretrovirals, antineoplastics, antifungals, antiparasitics, antianginals, rifamycin antibiotics, antiplatelets, selective estrogen receptor modulators, antidepressants, vinca alkaloids	rifampicin, efavirenz, nafcillin, rifabutin, rifapentine, flucloxacillin	clarithromycin, fosamprenamvir, etritonavir, ketoconazole, itraconazole, ritonavir, voriconazole, erythromycin, fluconazole, ciprofloxacin, norfloxacin
**CYP1A2**		
**Substrates**	**Inducers**	**Inhibitors**
agomelatine, duloxetine, clozapine, olanzapine, melatonin, propranolol, theophylline	rifampicin	ciprofloxacin, acyclovir, valacyclovir
**CYP2C9**		
**Substrates**	**Inducers**	**Inhibitors**
acenocumarol, celecoxib, meloxicam, fluvastatin, phenytoin	rifampicin	fluconazole, sulfametoxazole–trimethoprim, favipiravir
**CYP2C19**		
**Substrates**	**Inducers**	**Inhibitors**
omeprazole, pantoprazole, diazepam, voriconazole, clopidogrel, phenytoin	rifampicin	fluconazole, voriconazole

## Data Availability

Not applicable.
